# Beyond mid-thigh: Ultrasound assessment of hamstring muscle architecture at multiple muscle lengths in relation to strength

**DOI:** 10.1371/journal.pone.0361496

**Published:** 2026-10-27

**Authors:** Gulsah Karaca Yilmaz, Ali Cihan Dagli, Omer Batin Gozubuyuk, Nazif Ekin Akalan, Sergen Devran, Meryem Merve Oren Celik, Bulent Bayraktar

**Affiliations:** 1 Faculty of Behavioural and Movement Sciences, Musculoskeletal Physiotherapy Sciences, Vrije Universiteit Amsterdam, Amsterdam, Netherlands; 2 School of Health Sciences, University of Nottingham, Nottingham, United Kingdom; 3 Cyber-physical Health and Assistive Robotics Technologies Research Group, University of Nottingham, Nottingham, United Kingdom; 4 Back, Neck and Joint, Melbourne, Australia; 5 Istanbul Kültür University, Faculty of Health Sciences, Department of Physiotherapy and Rehabilitation, Istanbul, Turkey; 6 Istanbul Kültür University, Motion Analysis Centre, Istanbul, Turkey; 7 Istanbul University, Istanbul Faculty of Medicine, Department of Sports Medicine, Istanbul, Turkey; 8 Istanbul University, Istanbul Faculty of Medicine, Department of Public Health, Istanbul, Turkey; New Jersey Institute of Technology, UNITED STATES OF AMERICA

## Abstract

**Objectives:**

To investigate the relationship between ultrasound-based muscle architecture parameters and isometric knee flexor muscle strength in healthy individuals, and to evaluate whether distal (25%) and proximal (75%) measurement sites differ in their ability to reflect muscle strength compare to the commonly used mid-thigh (50%) region.

**Methods:**

Isometric muscle strength was measured in 20 healthy (22.15 ± 2.41 y; 8 females, 12 males) participants using a hand-held dynamometer. Ultrasound parameters (cross-sectional area, muscle thickness, pennation angle) of the biceps femoris-long head, semitendinosus, and semimembranosus were measured at 25%, 50%, and 75% of thigh length. Associations between strength and ultrasound-based parameters were examined using multiple linear regression analysis.

**Results:**

Moderate-to-high correlations (r = 0.615–0.815) were observed between muscle strength and cross-sectional area for all muscles at 50%. The regression model explained 89% of the variance in muscle strength (*p* < 0.001). Notably, the cross-sectional area of the semimembranosus and the total hamstring cross-sectional area at 25% length demonstrated strong correlations with muscle strength (r = 0.907 and 0.802, respectively). At 75%, the cross-sectional area of the semimembranosus showed a non-significant association with strength, while it could not be measured for the semitendinosus and biceps femoris-long head due to anatomical limitations. Muscle thickness showed significant correlations for the semitendinosus and semimembranosus (r = 0.702 and 0.698, respectively), whereas pennation angle demonstrated weaker and inconsistent relationships.

**Conclusions:**

The cross-sectional area of the semimembranosus at 50% of muscle length showed the strongest ultrasound-based predictor of isometric muscle strength. However, measurement at the distal site demonstrated robust associations, suggesting this region may serve as a practical alternative. In contrast, proximal measurements were limited and showed non-significant associations with strength. These findings can help expand the clinical and research applications of ultrasound imaging for assessing hamstring strength. Given the exploratory nature of this study, these findings should be interpreted cautiously.

## Introduction

Ultrasound (US) has become one of the widely used imaging techniques in both clinical and research settings for the assessment of muscle architecture, valued for its reliability, portability, and capacity for real-time dynamic evaluation [1]. Key US-derived parameters including muscle thickness (MT), cross-sectional area (CSA), and pennation angle (PA) serve as fundamental indicators of muscle architecture and are closely linked to functional capacity. Previous research has utilised US to characterise the architecture of the quadriceps [2,3] and gastrocnemius [4], whereas the hamstring muscles including the biceps femoris-long head (LBF), semitendinosus (ST), and semimembranosus (SM), have received comparatively little attention. Notably, only a limited number of studies have comprehensively examined all three hamstring muscles together [5].

Understanding hamstring architecture is clinically vital, as muscle strength is a primary determinant of functional health; muscle weakness is strongly related to functional limitations and physical disability [6]. In clinical practice, hand-held dynamometry (HHD) is a widely used valid and reliable tool for assessing muscle strength owing to its reasonable price and practicality in the healthy population [7,8]. Objective measurement of hamstring strength is crucial in many different fields concerned with musculoskeletal abnormalities, since hamstring strains are the most common muscle injury in athletes, often caused by insufficient hamstring strength [9]. Moreover, hamstring muscle strength measurement plays a key role in return-to-play decisions following injury [9,10]. Therefore, measuring isometric knee flexor muscle strength (IKFS), defined as the maximal voluntary isometric force produced by the knee‑flexor muscles, is significant for tailoring the treatment to targeted hamstring muscles. While conventional strength testing provides valuable information, combining it with imaging-based markers of muscle architecture can offer deeper insights into muscle capacity and injury risk.

US-based muscle measurements such as MT and CSA have shown the potential relationship with maximum muscle strength [11]. Moreover, evidence shows a strong correlation between quadriceps muscle strength and the CSA [12], MT, and PA [13]. However, this relationship has not been thoroughly investigated in hamstring muscles of healthy individuals. Furthermore, current US measurements rely predominantly on the mid-thigh measurement [14]. However, muscle architecture is non-uniform along the length of a muscle, and exclusive reliance on the mid-region may fail to capture these regional differences [15,16]. Consistent with this, studies in other muscle groups, such as the quadriceps and gastrocnemius, regional variations in muscle architecture along the muscle length have been previously investigated, highlighting the importance of site-specific assessments [17,18]. From a clinical perspective, the choice of measurement site may influence how the relationship between ultrasound findings and muscle strength is interpreted. In this context, investigating alternative measurement sites is important, as distal regions are more prone to acute strain injuries, while proximal adaptations may reflect chronic or degenerative processes [19]. However, it remains unclear whether measurements taken from different regions of the hamstring muscles reflect muscle strength in the same way. Evaluating these alternative sites may provide more clinically meaningful insights into hamstring structure and its relationship to strength, enhancing the diagnostic and rehabilitative utility of US.

Therefore, the aim of the present study was (1) to investigate whether US-based muscle architecture parameters (MS, CSA, PA) of the hamstring muscles correlate with maximum voluntary isometric strength, and (2) to examine whether distal (25%) and proximal (75%) measurement sites differ in their ability to reflect muscle strength comparison to the commonly used 50% site.

## Methods

### Study design

This cross- sectional study was conducted at the Department of Physiotherapy and Rehabilitation, Faculty of Health Sciences, Istanbul Kultur University, between August and June 2020. The study was prospectively registered at ClinicalTrials.gov (identifier: NCT04511143) and was approved by the local medical ethical committee (Approval No: 21/10/2019; 1291) in accordance with the principles of the 2018 Declaration of Helsinki. Written informed consent was obtained from all participants prior to study enrolment.

### Subjects

Twenty healthy participants (22.15 ± 2.41 y; 1.72 ± 0.09 m; 66.5 ± 11.50 kg; 8 females, 12 males) participated in the present study. The inclusion criteria were adults aged 18–30 years, who were willing and able to provide informed consent and perform the study procedures. Exclusion criteria included a history of musculoskeletal disorders, injuries, or surgeries involving the hip, knee, or ankle; neurological or systemic conditions affecting motor control; regular participation in competitive sports or high-intensity training programs, use of medications influencing muscle mass, neuromuscular function, or recovery (e.g., corticosteroids, muscle relaxants); pregnancy; and contradictions to US application.

### Procedures

The long head of the biceps femoris (LBF), ST, and SM were evaluated using B-mode US device (Toshiba Aplio 500, Japan) with a linear probe (10 − 12 MHz, with a footprint of 52 mm). Participants were asked to not make any changes to their activity levels during the three days prior to the assessment. All participants were placed in a relaxed prone position with 15° knee flexion for 10 minutes prior to evaluation [20]. Assessments were performed on the non-dominant extremity to reduce the influence of limb dominance, given the well-documented strength asymmetries between limbs that may affect measurement consistency [21,22]. All ultrasound measurements were performed by a sports physician with formal training in musculoskeletal ultrasound and over three years of experience in this field. A second experienced examiner independently performed repeated measurements for reliability analysis. The examiners were not blinded to the study but were not involved in the strength assessment or statistical analysis.

Reference points were marked at the ischial tuberosity and lateral/medial joint lines of the knee [16]. Subsequently, images were obtained at 25%, 50%, and 75% of this distance; medially for ST and SM, and laterally for LBF.

The CSA was measured in the transverse plane for ST (25% and 50%), SM (at 25%, 50%, and 75%), and LBF (25% and 50%). Only the SM was measured at the 75% level since at this point, muscle fibres were either absent (specifically for the ST muscle) or difficult to differentiate from those of the ST and LBF muscles. Total CSA was calculated as the sum of all three muscles at 25% and 50% levels (Fig 1).

**Fig 1:**
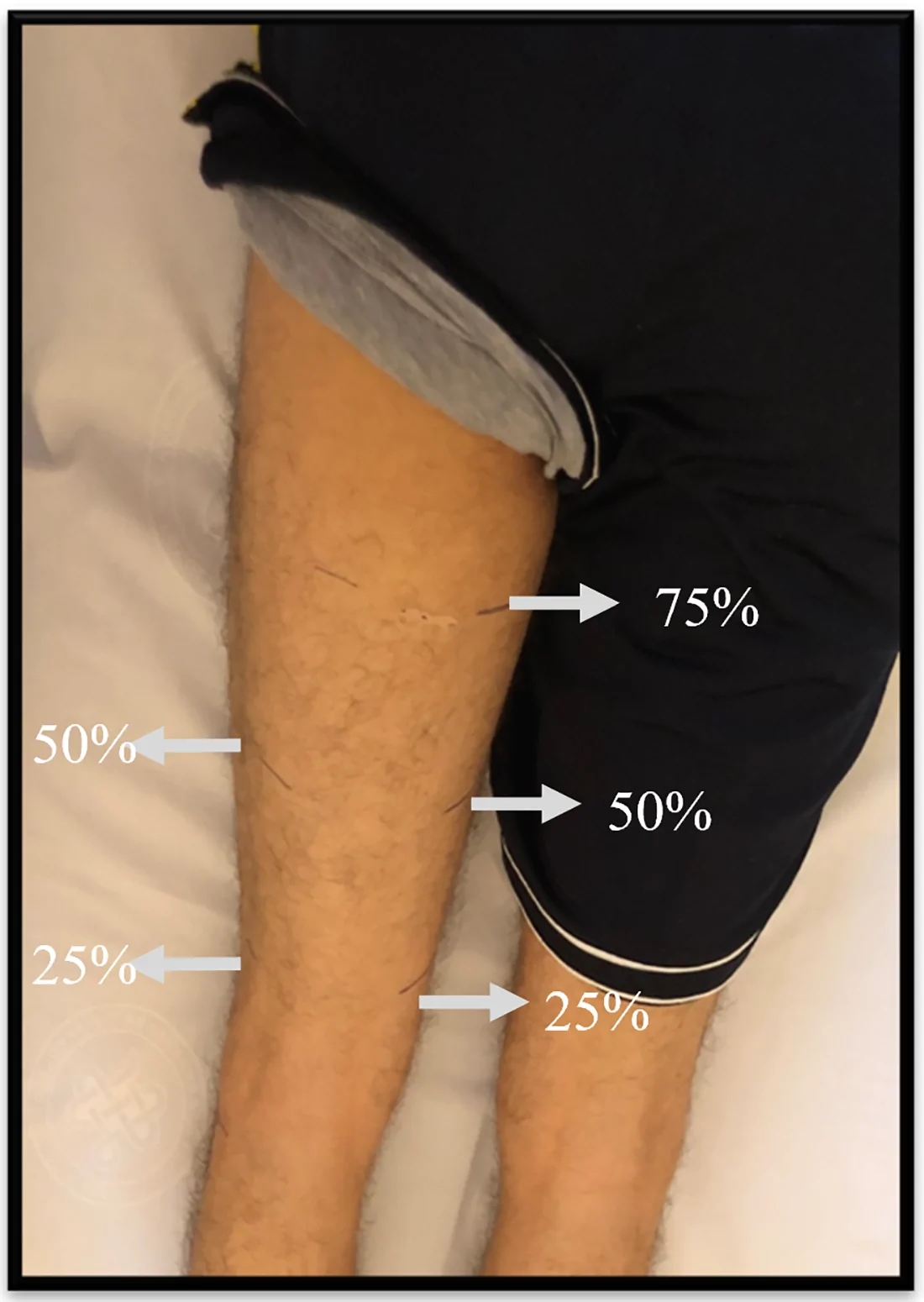
The CSA, PA, and MT measurement points on the ST, SM, and LBF muscles at different sites.

The probe was aligned longitudinally at 50% site to permit the measurement of the PA and MT [16,23]. PA was measured as the angle between the intermuscular and superficial aponeuroses for the SM and LBF muscles (Fig 2). In addition, a measurement was taken between the deep and superficial aponeuroses in the ST muscle due to a lack of intermuscular aponeurosis [16].

**Fig 2:**
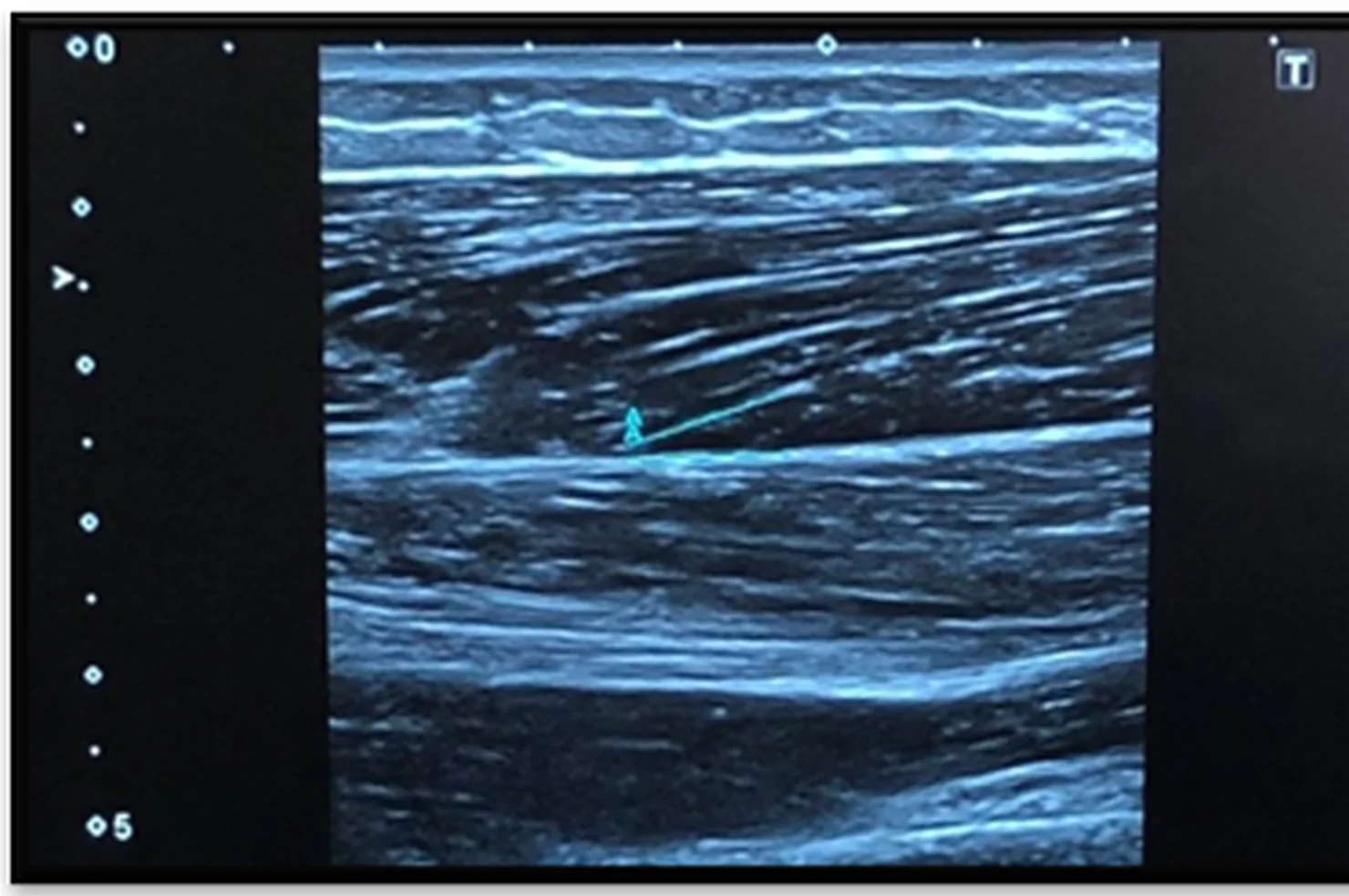
Longitudinal plane ultrasound image of LBF PA.

To assess inter-rater reliability, the intra-class correlation coefficient (ICC two-way random effects model, absolute agreement) was calculated based on measurements obtained by two independent evaluators in the first 11 participants. The intra-rater reliability of the first physician is shown elsewhere [24].

### Isometric muscle strength assessment

Maximum voluntary IKFS was measured on the non-dominant side using a hand-held dynamometer (HHD; Lafayette Model 01165) (Lafayette, IN) [25]. Participants were prone with 15° knee flexion with the foot and ankle located off the bed. The knee flexion was measured using a goniometer and maintained at 15° using an ankle strap to ensure consistent positioning (Fig 3). The HHD was placed 5 cm proximal to the medial malleolus using a stabilisation belt [26]. Participants performed three 5-second maximum voluntary isometric contractions; the average of these trials was used for analysis [26].

**Fig 3:**
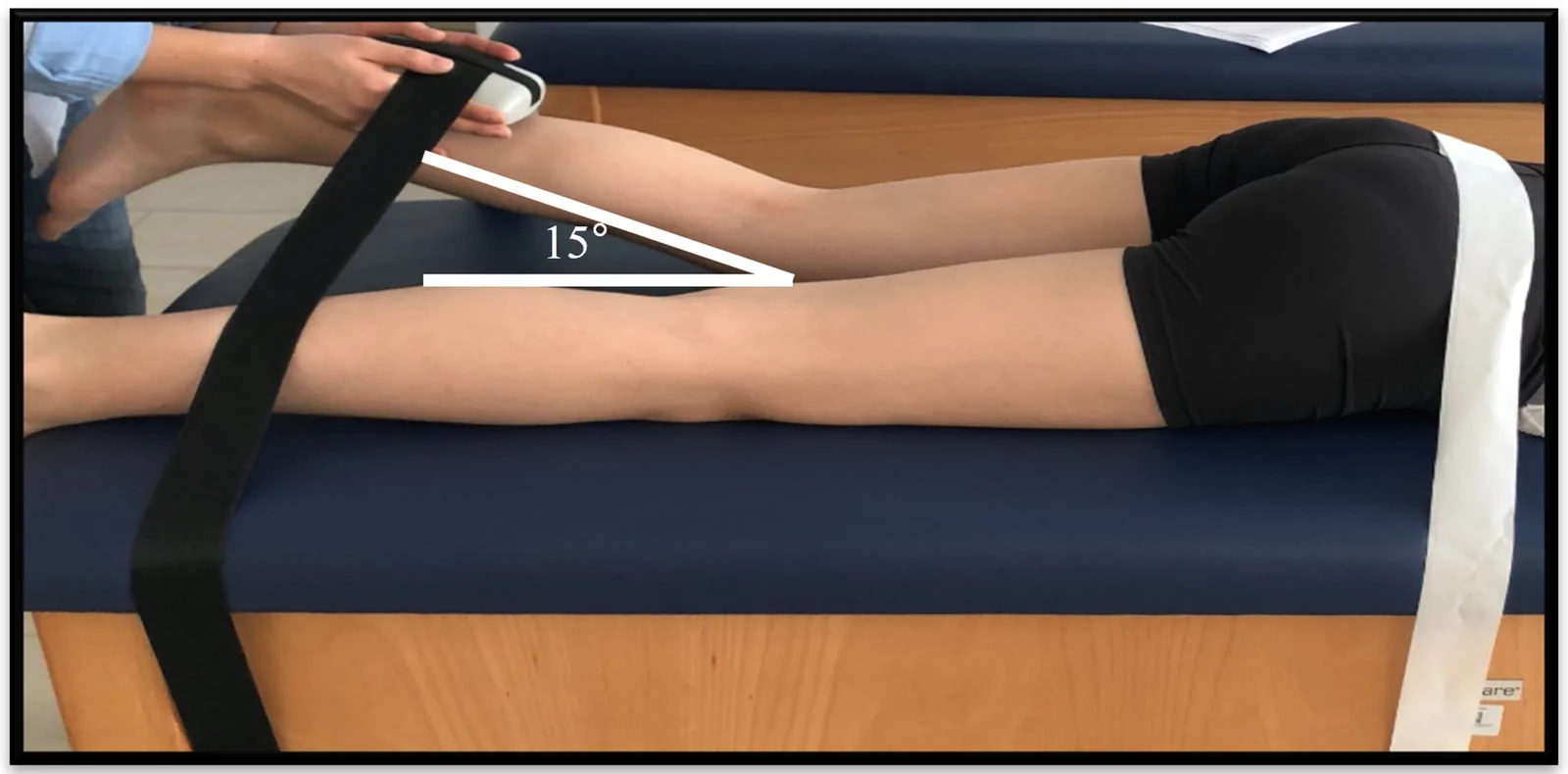
Isometric knee flexor muscle strength measurement.

### Inclusivity in global research

Additional information regarding the ethical, cultural, and scientific considerations specific to inclusivity in global research is included in the Supporting Information (S1 File).

### Statistical analyses

Sample size for the present study was determined using G*Power 3; 14 participants were required to achieve 95% power with an effect size of 0.73 [23]. To account for potential attrition, 20 participants were included in the present study. Data normality was confirmed via Shapiro-Wilk tests and visual inspection of histograms.

Descriptive statistics are reported as the mean ± standard deviation. The relationship between IKFS and US parameters was determined using Pearson’s or Spearman’s correlation coefficients (r), as appropriate based on data distribution. In addition, inter-rater reliability between the two evaluators was assessed using the intraclass correlation coefficient (ICC) in the first 11 participants [27]. To identify independent predictors of IKFS, a multiple linear regression model was analysed. Multicollinearity among the independent variables included in the multiple linear regression model was assessed using tolerance values, variance inflation factor (VIF), and condition index values. Tolerance values below 0.20, VIF values above 5, and condition index values above 30 were considered indicative of potentially problematic multicollinearity [28]. The model fit was assessed using appropriate residual statistics. An alpha level of *P* < 0.05 indicates statistical significance of the regression results. Statistical analyses were performed using SPSS v21.

A post hoc power analysis was conducted to evaluate the statistical power of the observed correlations between hamstring muscle architecture parameters (CSA, MT, PA) and IKFS. The observed effect sizes (Pearson’s r values), sample size, and a significance level of α = 0.05 (two-tailed) were used to calculate the achieved power (1 − β). This post hoc power analysis provided the probability that the study could detect the observed relationships, ensuring the robustness of the findings [29].

## Results

### Participants characteristics and reliability

The muscle architecture parameters, strength measurements, and participant demographics are detailed in Table 1. The demographic characteristics indicate that the sample consisted of young adults (age: 22.2 ± 2.4 y) with a mean body mass index (BMI) of 22.3 ± 2.6.

**Table 1:** Participant demographics; descriptive data for muscle architecture and the strength measures.

	Mean ± Std. Dev.	Minimum	Maximum
**Participant demographics**			
Age (y)	22.2 ± 2.4	18.0	27.0
Height (cm)	172.3 ± 9.1	158.0	190.0
Weight (kg)	66.5 ± 11.5	49.0	85.0
Body mass index (kg/m^2^)	22.3 ± 2.6	17.5	26.2
**Descriptive data**			
LBF CSA, 25% (cm^2^)	3.634 ± 1.904	1.580	8.633
ST CSA, 25% (cm^2^)	1.111 ± 0.734	0.340	3.280
SM CSA, 25% (cm^2^)	9.474 ± 2.904	5.31	15.45
LBF CSA, 50% (cm^2^)	9.583 ± 1.940	6.670	14.057
ST CSA, 50% (cm^2^)	5.082 ± 1.955	2.957	9.400
SM CSA, 50% (cm^2^)	9.644 ± 1.888	5.825	12.687
LBF MT, 50% (mm)	29.087 ± 5.109	21.350	44.500
ST MT, 50% (mm)	24.393 ± 4.315	16.200	33.167
SM MT, 50% (mm)	30.488 ± 4.367	22.900	37.667
LBF PA, 50% (°)	12.150 ± 3.180	3.333	16.333
ST PA, 50% (°)	4.125 ± 2.869	1.666	12.666
SM PA, 50% (°)	14.208 ± 3.645	6.500	19.500
SM CSA, 75% (cm^2^)	2.817 ± 1.253	0.76	6.283
Isometric Knee Flexion Strength (N)	247.082 ± 81.169	102.967	366.767
Isometric Knee Flexion Strength – female (N)	165 ± 50.800	102.967	275.366
Isometric Knee Flexion Strength – male (N)	301.8 ± 39.600	254.366	366.767

**Abbreviations:** LBF = Biceps Femoris-Long Head; ST = Semitendinosus; SM = Semimembranosus; CSA = Cross-Sectional Area; MT = Muscle Thickness; PA = Pennation Angle; 25%, 50%, or 75% = 25%, 50%, or 75% of the distance between the tuber ischiadicum and the knee.

Inter-rater reliability for the CSA, MT, and PA are summarised in Table 2. Reliability analysis shows that the CSA, MT, and PA measurements were highly consistent across the LBF, ST, and SM between the two evaluators, with ICCs ranging from 0.878 to 0.999.

**Table 2:** Inter-rater coefficient for US-based muscle architecture parameters.

Parameters	Inter-rater Coefficient (95% Cl) (n = 11)
LBF CSA, 25% (cm^2^)	0.999 (0.998-1.00)
ST CSA, 25% (cm^2^)	0.982 (0.935-0.995)
SM CSA, 25% (cm^2^)	0.962 (0.864-0.990)
LBF CSA, 50% (cm^2^)	0.949 (0.819-0.986)
ST CSA, 50% (cm^2^)	0.994 (0.977-0.998)
SM CSA, 50% (cm^2^)	0.878 (0.569-0.967)
LBF MT, 50% (mm)	0.966 (0.879-0.991)
ST MT, 50% (mm)	0.990 (0.964-0.997)
SM MT, 50% (mm)	0.990 (0.966-0.997)
LBF PA, 50% (°)	0.974 (0.909-0.993)
ST PA, 50% (°)	0.961 (0.864-0.989)
SM PA, 50% (°)	0.935 (0.771-0.982)
SM CSA, 75% (cm^2^)	0.966 (0.879-0.991)

**Abbreviations:** CSA = Cross-Sectional Area; LBF = Biceps Femoris-Long Head; MT = Muscle Thickness; PA = Pennation Angle; SM = Semimembranosus; ST = Semitendinosus; 25%, 50%, or 75% = 25%, 50%, or 75% of the distance between the tuber ischiadicum and the knee.

### Correlations between US parameters and strength

Correlation analysis between US parameters and 15° IKFS revealed several significant relationships. Regarding CSA, strong correlations were observed for the SM at both 25% (*P* < 0.01; r = 0.907) and at 50% (*P* < 0.01; r = 0.911). The ST showed a significant association at 50% (*P* < 0.01; r = 0.802),while its correlation at 25% was not significant (P = 0.327; r = 0.231). Similarly, the LBF demonstrated a moderate correlation at 50% (P < 0.01; r = 0.615), whereas no significant correlation was found at 25% (P = 0.248; r = 0.271). The total CSA demonstrated strong correlations at both 25% (P < 0.01; r = 0.802) and 50% (P < 0.01; r = 0.911). In contrast, CSA at 75% (SM) did not show a significant correlation with strength (P = 0.217; r = −0.289) (Table 3).

**Table 3:** Correlations between US-based muscle architecture parameters and muscle strength.

Parameters	15° IKFS (N)	
	(n = 20)	
	p values	r values
**CSA (cm**^**2**^)		
**LBF, 25%**	0.248 ^(β)^	0.271
**ST, 25%**	0.327 ^(β)^	0.231
**SM, 25%**	< 0.01^(α)^	0.907**
**Total, 25%**	<0.01^(β)^	0.802**
**LBF, 50%**	<0.01^(β)^	0.615**
**ST, 50%**	<0.01^(β)^	0.802**
**SM, 50%**	< 0.01^(α)^	0.815**
**Total, 50%**	< 0.01^(α)^	0.911**
**SM, 75%**	0.217 ^(β)^	−0.289
**MT (mm)**		
**LBF, 50%**	0.313 ^(β)^	0.238
**ST, 50%**	< 0.01^(α)^	0.702**
**SM, 50%**	< 0.01^(α)^	0.698**
**PA (°)**		
**LBF, 50%**	< 0.05 ^(β)^	0.451*
**ST, 50%**	0.744 ^(β)^	0.078
**SM, 50%**	< 0.05 ^(β)^	0.524*

*Correlation is significant at the 0.05 level.

**Correlation is significant at the 0.01 level.

(α) Pearson’s coefficient tests.

(β) Spearman’s coefficient tests.

(θ) Student T-test.

**Abbreviations:** CSA = Cross-Sectional Area; IKFS = Isometric knee flexor muscle strength; LBF = Biceps Femoris-Long Head; MT = Muscle Thickness; PA = Pennation Angle; SM = Semimembranosus; ST = Semitendinosus; 25%, 50%, or 75% = 25%, 50%, or 75% of the distance between the tuber ischiadicum and the knee.

For muscle thickness (MT), significant correlations with IKFS were found for the ST (P < 0.01; r = 0.702) and SM (P < 0.01; r = 0.698), whereas no significant correlation was observed for the LBF (P = 0.313; r = 0.238).

For pennation angle (PA), correlations were generally weaker and more variable. The SM showed a moderate correlation with IKFS (P < 0.05; r = 0.524), and the LBF demonstrated a weak but significant correlation (P < 0.05; r = 0.451). No significant correlation was found for the ST (P = 0.744; r = 0.078) (Table III).Additionally, the differences in strength between males and females were also statistically significant (*P* < 0.01).

### Regression analysis and power

A multiple linear regression was performed to identify independent predictors of IKFS (Table 4) (Fig 4). This model was statistically significant (*P* < 0.001), explaining 89% of the variance in IKFS (r^2^ = 0.89). The linear regression equation was:


$$\begin{array}{c} \text{IKFS}:\;-\text{25}.\text{28}+\text{3}.\text{152 }(\text{LBF CSA},\text{50}\%)+\text{12}.\text{978 }(\text{ST CSA},\text{50}\%)+\text{14}.\text{573 }(\text{SM CSA},\text{50}\%)+\text{59}.\text{428 }(\text{sex}) \end{array}$$

**Table 4:** A multiple linear regression model for predictors of IKFS.

	B	B Std. Error	Beta	p	Tolerance	VIF
**(Constant)**	−25.280	55.318		0.654		
**LBF CSA, 50% (cm2)**	3.152	5.193	0.075	0.553	0.474	2.110
**ST CSA, 50% (cm2)**	12.978	4.990	0.313	0.020	0.506	1.978
**SM CSA, 50% (cm2)**	14.573	5.203	0.339	0.013	0.499	2.005
**Sex**	59.428	23.264	0.368	0.022	0.352	2.841

**Abbreviations:** CSA = Cross-Sectional Area; IKFS = Isometric knee flexor muscle strength; LBF = Biceps Femoris-Long Head; MT = Muscle Thickness; PA = Pennation Angle; SM = Semimembranosus; ST = Semitendinosus; 25%, 50%, or 75% = 25%, 50%, or 75% of the distance between the tuber ischiadicum and the knee.

**Fig 4:**
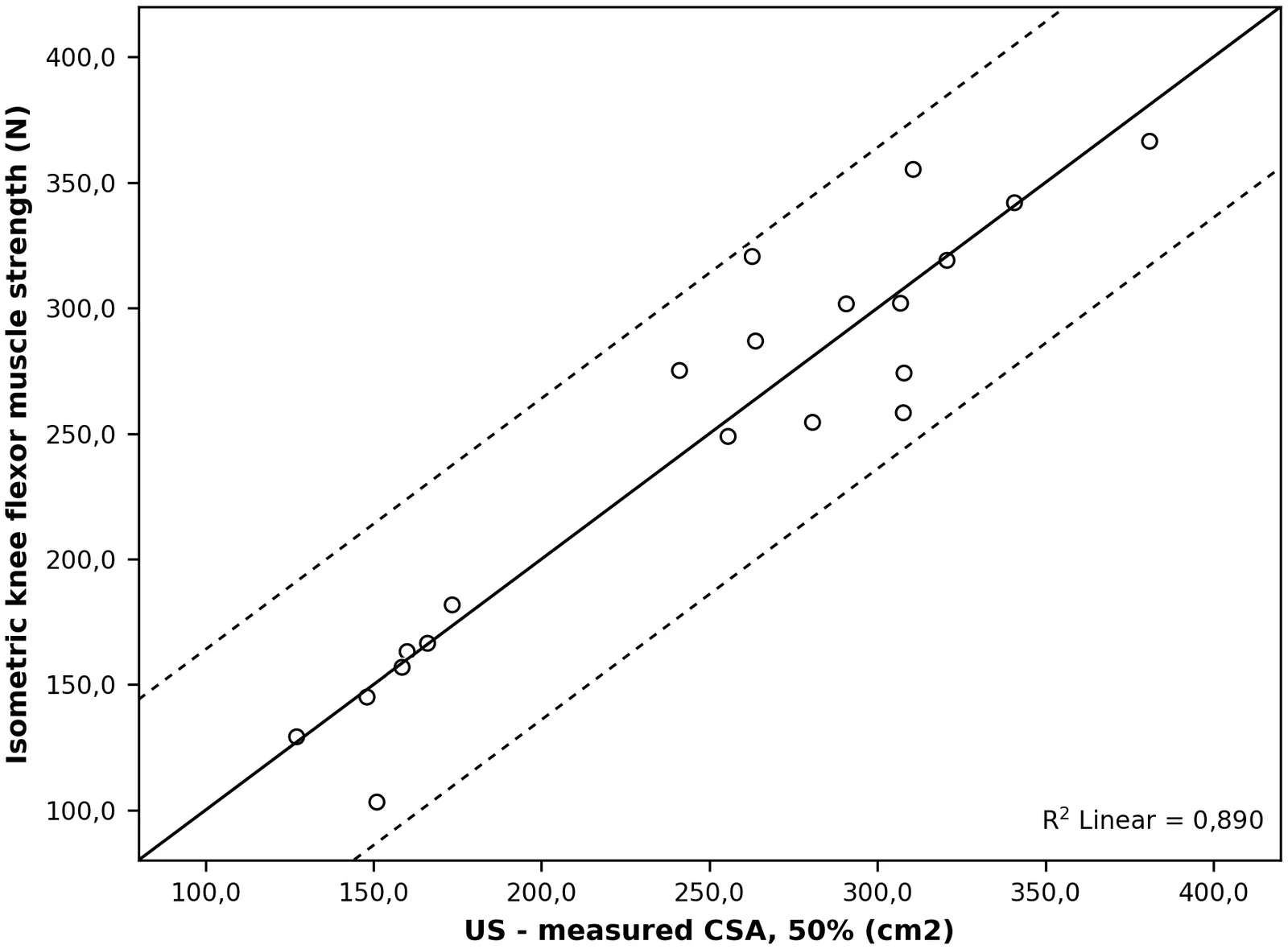
Multiple linear regression analysis. Scatterplots and multiple linear regression lines indicating correlations between Isometric knee flexor muscle strength and ultrasound-measured CSA, 50%. Lines of best fit (solid line) with 95% confidence intervals (dashed lines) are indicated in the graphs where significant relationships were assigned.

Multicollinearity diagnostics did not indicate problematic collinearity among the predictors included in the final regression model. Tolerance values ranged from 0.352 to 0.506, and VIF values ranged from 1.978 to 2.841. The maximum condition index was 23.730. These findings indicate that, although the CSA values of the synergistic hamstring muscles were correlated, the degree of collinearity was not severe enough to compromise the stability of the regression model.

Post-hoc power analysis revealed that the observed effect sizes (r = 0.615 to 0.911) yielded a statistical power between 0.81 and 0.94. These results indicate that the study was adequately powered to detect the observed relationships between US-based muscle architecture parameters and IKFS.

## Discussion

The primary finding of this study is that US-derived muscle architecture, particularly CSA of the SM and the total hamstring CSA at 25 and 50%, and the MT of the SM at 50% are strongly correlated with maximum voluntary IKFS. Notably, the strong correlations were observed at the distal 25% for the SM (r = 0.907) and the 50% site for the total CSA (r = 0.911),), highlighting the importance of both distal and mid-thigh regions. Although total CSA showed the strongest correlation with IKFS, individual muscle CSAs were used in the regression model to identify muscle-specific contributions and to avoid overlap between the combined and individual measures. These findings indicate that the relationship between muscle architecture and strength varies along the muscle length. In addition to the focus on the mid-thigh (50%) region, distal measurement sites (25%), particularly for CSA of the semimembranosus and total CSA, may also provide valuable insight into muscle strength. In contrast, measurements at the proximal region (75%) showed weaker associations, suggesting limited predictive value for strength. Collectively, these results emphasise the importance of multi-site assessment to capture regional muscular variations in muscle morphology and improve the accuracy of strength estimation [1,4]. This has practical implications for both clinical assessment and research, offering greater flexibility in selecting measurement sites that are reliable and reflective of functional strength.

In the present study, isometric hamstring strength was positively correlated with the CSA and MT of the SM, ST, and LBF, consistent with the general principle that physiological CSA is related to a muscle’s force-generating capacity [30,31]. The physiological basis for this relationship is that a larger CSA reflects a greater number of sarcomeres, and force production scales with the amount of contractile tissue available to generate tension [30]. Although similar associations have been reported in older adults and clinical populations, our results extend this relationship to a younger, healthy cohort, indicating that muscle architectural characteristics play a consistent role in strength across populations [3,4]. However, in clinical conditions such as hamstring strains, where pain and tenderness are common, muscle strength may not be objectively assessed using HHD or isokinetic testing due to painful contractions [32,33]. Utilising US-based indirect muscle strength measurement may provide some insight into muscle strength “without pain,” which is important when returning to sports following hamstring injury [10]. However, it should be noted that muscle strength is multifactorial, and neural activation, tendon properties, and muscle fibre composition may also contribute to the observed variability. Therefore, while CSA and MT appear to be important determinants, they should be considered alongside other neuromuscular factors when interpreting hamstring strength capacity.

The balance of muscle strength between the anterior and posterior thigh muscles is a fundamental factor for maintaining motor control and functional performance. The observed relationship between hamstring strength and US-derived architectural parameters (CSA and MT) in this study suggests that morphological characteristics provide valuable insight into functional muscle capacity. Similar associations have been also reported in other muscle groups, such as the quadriceps, where greater muscle thickness and CSA are linked to higher force production [12,13,34]. This supports the notion that muscle architecture plays a central role in determining strength across different anatomical regions. In this context, US muscle architecture parameters may offer a non-invasive approach to estimate isolated muscle in neurologically affected patients. Although the current study evaluated US parameters in healthy participants, the findings provide a necessary baseline for understanding how architectural parameters relate to isolated muscle force. Therefore, establishing the relationship between isometric strength and US-derived muscle architecture in healthy individuals is an essential step before examining these associations in clinical populations.

Interestingly, the regression analysis in this current study identified the SM has the most significant relationship with strength. Furthermore, the LBF had the lowest coefficient in the model. This discrepancy may be explained by the nature of the testing of this study that isometric assessment was used at 15° of the knee flexion, since most injuries occur during eccentric contraction at longer muscle length [35]. On the other hand, the CSA is not directly associated with the risk of injury [35,36], despite evidence of a positive correlation between the CSA and muscle strength [37,38]. This distinction is particularly important, as the present study evaluated muscle strength under isometric conditions at a fixed joint position, whereas hamstring injuries are typically associated with eccentric loading at longer muscle lengths [35]. Therefore, these findings should be interpreted with caution in a clinical context, as CSA should not be used alone to assess for hamstring injury risk. While hamstring hypertrophy (increased CSA) remains a target for improving muscle strength, clinicians should exercise caution when using CSA alone to screen for injury vulnerability.

CSA measurements are typically obtained at mid-thigh (50%) level [5,35,39]. However, assessing all three muscles (ST, SM, and LBF) at a single point may not fully represent their morphological variability along the muscle length [39]. Therefore, to investigate this variability, CSA parameters were measured at 3 different levels (25%, 50%, 75%) in the present study. Despite high reliability metrics (ICC), some of the parameters could not be included due to anatomical limitations, such as the CSA at 75% of the ST and LBF, specifically the inability to obtain reliable CSA measurements at 75% for the ST and LBF because their muscle fibres either taper substantially or become difficult to differentiate from surrounding structures at this region. Additionally, no correlation was found between the CSA at 75% of the SM and IKFS (r = −0.29, *P* = 0.22).

In terms of measurement position, although 90° knee flexion is commonly preferred for the strength assessment, evidence suggests that hamstring EMG activity is higher at more extended angles (15 − 30°) than at more flexed positions (30 − 120°) [40]. Furthermore, strong correlations have been reported between knee muscle strength measurements obtained at 15° of flexion, indicating that assessment at a more extended knee angle provides valid and comparable outcomes [40,41]. From a methodological perspective, US imaging of the hamstrings is performed with the knee in a near-extended knee position to optimise probe access and minimise muscle shortening. Therefore, selecting 15° of knee flexion for isometric strength testing aligns the joint angle used for US imaging and strength measurement. This alignment likely improves the interpretability of the relationship between US-derived parameters such as CSA and functional outcomes such as maximal isometric strength. Moreover, adopting a standardised joint position that is both feasible for imaging and physiologically relevant for strength assessment may improve reproducibility across studies and facilitate future comparisons in populations with neuromuscular impairments.

This study has some limitations. Participants’ physical activity levels were not formally assessed; however, based on their background, they were neither engaged in structured athletic training nor completely sedentary. BMI was also not controlled during recruitment, which could theoretically influence US image quality due to subcutaneous fat thickness, although the mean BMI of the participants fell within in the normal weight classification [42].

Additionally, although sex contributed substantially to the regression model (β = 0.368), its inclusion should be interpreted with caution. The study was not specifically powered to detect sex-specific effects, and therefore the role of sex in the model is best understood as a general covariate to account for baseline biological variability rather than as a definitive predictor. Therefore, the coefficient for sex should not be interpreted as evidence of a detailed sex-specific mechanism.

Another limitation is that muscle strength was assessed only under isometric conditions, without the inclusion of isokinetic measurements. Isokinetic assessment could provide additional insight into muscle performance across different angular velocities and may better reflect dynamic functional capacity. Age‑related changes in muscle architecture and neuromuscular function, differences in training status, and variations in body composition may substantially alter the relationships observed here.

Finally, the relatively small sample size and the inclusion of only young, healthy adults limit the generalisability of the findings. Therefore, the results should be considered exploratory and may not directly translate to older individuals, clinical populations, or athletic cohorts. Future studies with larger and more diverse samples are needed to confirm and extend these observations. Future work should also incorporate eccentric strength outcomes, which may better reflect functional hamstring demands and injury‑relevant loading. Validation of ultrasound‑derived architectural measures against reference standards such as MRI or elastography would strengthen confidence in the accuracy and clinical applicability of these findings.

## Conclusion

US-based measurements of hamstring muscle architecture, particularly CSA, are strongly correlated with IKFS in healthy individuals. While the mid-thigh (50%) region remains a reliable site, our findings demonstrate that the distal region (25%) especially the CSA of the SM also serves as a highly reliable and valid alternative measurement site. In contrast, proximal measurements (75%) were limited by anatomical constraints and showed weak or non-significant associations with strength. These results highlight the value of assessing multiple sites along the muscle to improve the accuracy and flexibility of US evaluations. The findings also suggest that the relationship between muscle architecture and strength may vary depending on the measurement site. Incorporating measurements at both distal and mid-thigh sites may improve the accuracy of rehabilitation monitoring and provide a non-invasive, pain-free method for estimating muscle capacity although these findings are exploratory and require validation in clinical, older, and athletic populations.

## Supporting information

S1 File Inclusivity in Global Health Questionnaire. (PDF)

S2 File All data. (XLSX)
