# Supplementary material for: Beyond mid-thigh: Ultrasound assessment of hamstring muscle architecture at multiple muscle lengths in relation to strength
Source: PLoS One. 2026 Oct 27;21(10):e0361496. doi: 10.1371/journal.pone.0361496 (PMC13712582; doi:10.1371/journal.pone.0361496)
Supplement: Supplementary file 2 [file pone.0361496.s003.docx]

We sincerely thank you for your interest in and valuable contribution to our manuscript. We have carefully revised the manuscript in line with the reviewers’ comments and suggestions. Our point-by-point responses, along with the corresponding revisions in the manuscript, are detailed below.

We appreciate the opportunity to improve our work and look forward to your further feedback and consideration for publication in your journal.

Sincerely,

**Ali Cihan Dagli**

PhD Physiotherapy

School of Health Sciences

The University of Nottingham, UK

alyad2@nottingham.ac.uk

**RESPONSE TO EDITOR**

**Comment 1.**

Please ensure that your manuscript meets PLOS ONE's style requirements, including those for file naming. The PLOS ONE style templates can be found at

https://journals.plos.org/plosone/s/file?id=wjVg/PLOSOne_formatting_sample_main_body.pdf and

https://journals.plos.org/plosone/s/file?id=ba62/PLOSOne_formatting_sample_title_authors_affiliations.pdf

**Response:**

We would like to sincerely thank the editor/reviewer for this helpful comment.

We confirm that we have reviewed PLOS ONE’s style requirements, including file naming conventions, and have updated the manuscript and all associated files to ensure full compliance.

**Comment 2.**

Please include a complete copy of PLOS’ questionnaire on inclusivity in global research in your revised manuscript. Our policy for research in this area aims to improve transparency in the reporting of research performed outside of researchers’ own country or community. The policy applies to researchers who have travelled to a different country to conduct research, research with Indigenous populations or their lands, and research on cultural artefacts. The questionnaire can also be requested at the journal’s discretion for any other submissions, even if these conditions are not met. Please find more information on the policy and a link to download a blank copy of the questionnaire here: https://journals.plos.org/plosone/s/best-practices-in-research-reporting. Please upload a completed version of your questionnaire as Supporting Information when you resubmit your manuscript.

**Response:**

We would like to thank the editor for this helpful comment. We confirm that we have completed the PLOS questionnaire on inclusivity in global research and included it as Supporting Information in the revised submission, in accordance with the journal’s requirements.

**Comment 3.**

Please provide a complete Data Availability Statement in the submission form, ensuring you include all necessary access information or a reason for why you are unable to make your data freely accessible. If your research concerns only data provided within your submission, please write "All data are in the manuscript and/or supporting information files" as your Data Availability Statement.

**Response:**

We would like to thank for this helpful comment. We confirm that a Data Availability Statement has been included in the revised submission, stating: “All relevant data are within the paper and its Supporting Information files.” The relevant data have been provided as an Excel (.xlsx) file in the Supporting Information.

**Comment 4.**

Please amend either the abstract on the online submission form (via Edit Submission) or the abstract in the manuscript so that they are identical.

**Response:**

Thanks for highlighting this helpful comment. We confirm that we have amended the abstract to ensure that the version in the online submission form and the manuscript are now identical.

**Comment 5.**

We note that Figures 1a and 2 in your submission contain copyrighted images. All PLOS content is published under the Creative Commons Attribution License (CC BY 4.0), which means that the manuscript, images, and Supporting Information files will be freely available online, and any third party is permitted to access, download, copy, distribute, and use these materials in any way, even commercially, with proper attribution. For more information, see our copyright guidelines: http://journals.plos.org/plosone/s/licenses-and-copyright.

We require you to either (1) present written permission from the copyright holder to publish these figures specifically under the CC BY 4.0 license, or (2) remove the figures from your submission:

1. You may seek permission from the original copyright holder of Figures 1a and 2 to publish the content specifically under the CC BY 4.0 license.

**Response:**

We would like to kindly confirm that Figures 1a and 2 are original figures created by the authors specifically for this manuscript to illustrate the experimental setup and do not contain any previously published or copyrighted material. Therefore, we believe that no permission for reuse is required.

**Comment 6.**

Please include captions for your Supporting Information files at the end of your manuscript, and update any in-text citations to match accordingly. Please see our Supporting Information guidelines for more information: http://journals.plos.org/plosone/s/supporting-information.

**Response:**

We would like to thank the editor for this helpful comment. We confirm that we have included captions for all Supporting Information files at the end of the manuscript and have updated the in-text citations accordingly to ensure consistency with PLOS ONE Supporting Information guidelines.

**REVIEWER 1 SUGGESTIONS**

**Comment 1.**

Framing of the 75% Measurement Site

In the abstract and introduction, the authors propose exploring both the 25% and 75% sites as reliable alternatives to the mid-thigh measurement. However, the manuscript later reveals that reliable CSA measurements at 75% could not be obtained for the ST and LBF due to anatomical challenges (muscle fibers tapering and blending). Furthermore, the SM at 75% showed a weak, non-significant correlation with strength (r = -0.289). The abstract and introduction should be revised to remove the implication that the 75% site is a viable alternative, and instead accurately frame it as a site where proximal ultrasound measurements are anatomically challenging and poorly correlated with strength.

Response:

Thank you for this valuable contribution. We appreciate the opportunity to clarify this point. We agree that, in its original form, the abstract and introduction could be interpreted as suggesting that both the 25% and 75% sites may serve as alternative measurement regions. We have revised both the abstract and introduction to more accurately reflect our findings.

Our results indicate that the 75% site presents important anatomical limitations and does not demonstrate a meaningful association with strength. To improve clarity, we have revised the abstract and introduction. The relevant sentence in the abstract has been updated as follows:

[Line 25-27]

*“…to evaluate whether distal (25%) and proximal (75%) measurement sites differ in their ability to reflect muscle strength compared to the commonly used mid-thigh (50%) region.”*

[Line 39-41]

*“…At 75%, CSA showed a weak, non-significant association with strength for the semimembranosus, while it could not be measured for the semitendinosus and biceps femoris long head due to their indistinguishable structure…”*

In addition, the Conclusions section has been modified to better reflect our findings:

[Line 382-384]

*“…In contrast, proximal measurements were limited by anatomical constraints and showed weak or non-significant associations with strength. …”*

Similarly, the aim of the study in the Introduction has been revised to avoid presenting both regions as equivalent alternatives. These revisions ensure that the 75% site is now accurately framed as anatomically challenging and not suitable as a reliable alternative measurement site.

**Comment 2.**

Statistical Analysis & Regression Model Diagnostics

The multiple linear regression model explains 89% of the variance in IKFS, incorporating the 50% CSA of the individual LBF, ST, and SM muscles, as well as "Sex," as predictors.

* Multicollinearity: Because the sizes of synergistic muscles within the same individual are typically highly correlated, there is a high risk of multicollinearity in this model. The authors must explicitly report testing for multicollinearity (e.g., Variance Inflation Factor [VIF] or tolerance values) to ensure these independent predictors are not improperly inflating the model's predictive power.

* The "Sex" Variable: The regression model relies heavily on the "Sex" variable (Beta = 0.368). However, the authors explicitly state in their limitations that the study was "not powered to detect detailed sex-specific effects". The authors must clarify the statistical justification for heavily weighting an underpowered variable in the primary predictive model.

Response:

We thank the reviewer for this important methodological comment. We agree that including the CSA values of anatomically and functionally related hamstring muscles in the same regression model may raise concerns regarding multicollinearity. We’ve addressed both issues based on the comments as follows:

Multicollinearity

In response, we performed formal multicollinearity diagnostics for all predictors included in the multiple linear regression model and added these results to the revised manuscript.

The tolerance values ranged from 0.352 to 0.506, and the variance inflation factor (VIF) values ranged from 1.978 to 2.841. These values were within commonly accepted limits, indicating that problematic multicollinearity was not present in the final regression model. In addition, the maximum condition index was 23.730, which was below the commonly used threshold of 30 for severe multicollinearity. Therefore, although the CSA values of synergistic hamstring muscles were expected to be correlated, the observed degree of collinearity was not sufficient to invalidate the regression estimates or to suggest that the model’s explanatory performance was artificially inflated by multicollinearity.

We have revised the Statistical Analyses section to specify that multicollinearity was assessed using tolerance, VIF, and condition index values. We have also added the tolerance and VIF values to Table IV and included the collinearity diagnostics in the Results section. In addition, we revised the interpretation of the regression findings to avoid overemphasizing the isolated independent contribution of each individual muscle. The model is now interpreted primarily as an explanatory model for IKFS rather than as evidence of separate causal effects of each muscle.

[Line 176-180]

*“…Multicollinearity among the independent variables included in the multiple linear regression model was assessed using tolerance values, variance inflation factor (VIF), and condition index values. Tolerance values below 0.20, VIF values above 5, and condition index values above 30 were considered indicative of potentially problematic multicollinearity…”*

[Line 250-255]

*“…Multicollinearity diagnostics did not indicate problematic collinearity among the predictors included in the final regression model. Tolerance values ranged from 0.352 to 0.506, and VIF values ranged from 1.978 to 2.841. The maximum condition index was 23.730. These findings indicate that, although the CSA values of the synergistic hamstring muscles were correlated, the degree of collinearity was not severe enough to compromise the stability of the regression model…”*

[Line 245-249]

***Table IV.*** *A multiple linear regression model for predictors of IKFS*

*Two columns for tolerance and VIF were added in Table IV.*

The “Sex” variable

Regarding the inclusion of sex in the model, we agree that the present study was not powered to perform detailed sex-specific analyses or to test sex-based effect modification. However, sex was included as an adjustment covariate rather than as a primary explanatory variable. This decision was based on the known differences between males and females in muscle size, body composition, and strength. Therefore, omitting sex from the model could have introduced confounding in the association between hamstring CSA and IKFS.

We have clarified in the revised Discussion and Limitations sections that the coefficient for sex should not be interpreted as evidence of a detailed sex-specific mechanism. Rather, sex was retained in the model to adjust for systematic between-sex differences in muscle size and strength. We have also emphasised that future studies with larger samples are required to examine sex-specific associations and sex-by-muscle CSA interaction.

[Line 357-362]

*“…Additionally, although sex contributed substantially to the regression model (β = 0.368), its inclusion should be interpreted with caution. The study was not specifically powered to de-tect sex-specific effects, and therefore the role of sex in the model is best understood as a general covariate to account for baseline biological variability rather than as a definitive pre-dictor. Therefore, the coefficient for sex should not be interpreted as evidence of a detailed sex-specific mechanism.…”*

**Comment 3.**

Data Availability (PLOS ONE Policy Compliance)

In the Data Availability statement, the authors note that "All relevant data are within the manuscript". However, only summary statistics (means, standard deviations, correlation coefficients) are provided in the manuscript. PLOS ONE policy explicitly requires that the raw data points behind means, medians, and variance measures be fully available without restriction. The authors must provide the raw dataset (e.g., the individual strength and ultrasound metrics for the 20 participants) as a supplementary file or deposit it in a public repository, unless specific legal/ethical restrictions apply.

**Response:**

We would like to thank for this helpful comment. We confirm that a Data Availability Statement has been included in the revised submission, stating: “All relevant data are within the paper and its Supporting Information files.” The relevant data have been provided as an Excel (.xlsx) file in the Supporting Information.

**Comment 4.**

Clinical Context of Isometric vs. Eccentric Strength

The authors appropriately note that the LBF is the most common site for hamstring strains, which typically occur during eccentric contractions at longer muscle lengths. Because this study measures isometric strength at 15° of knee flexion, the LBF showed a lower correlation with strength compared to the SM. The authors should briefly expand the discussion to caution clinicians against relying solely on CSA to screen for injury vulnerability, explicitly noting that CSA is not directly associated with the risk of injury.

Response:

Thank you for this important and clinically relevant suggestion. In line with your comment, we have expanded the Discussion to better clarify the distinction between the isometric strength assessment used in this study and the eccentric loading conditions under which hamstring injuries typically occur. Specifically, we added the following sentences to highlight this difference and improve the clinical interpretation of our findings:

[Line 321-325]

*“…This distinction is particularly important, as the present study evaluated muscle strength under isometric conditions at a fixed joint position, whereas hamstring injuries are typically associated with eccentric loading at longer muscle lengths [37]. Therefore, these findings should be interpreted with caution in a clinical context, as CSA should not be used alone to assess for hamstring injury risk.”*

In addition, we explicitly emphasise that CSA should not be interpreted as a direct indicator of injury risk and that clinicians should exercise caution when relying solely on CSA measurements to screen for injury vulnerability. We believe these revisions strengthen the clinical relevance and interpretation of the results.

**Comment 5.**

5) Generalizability

The study's cohort consists of 20 healthy young adults. The conclusion should more explicitly remind readers that age-related changes, training status, and musculoskeletal pathologies can substantially alter muscle-strength associations. Therefore, these findings are exploratory and require validation in clinical, older, or athletic populations.

Response: We appreciate this insightful suggestion. In line with your suggestion, we have revised the Conclusion to include a clearer statement regarding the scope of our findings. While this aspect was addressed in the Discussion, we agree that it should also be explicitly stated in the Conclusion.

Specifically, we added a statement clarifying that the findings are exploratory and require validation in broader populations, including clinical, older, and athletic groups [line 381-385].

**Minor Comments:**

**Comment 1.**

* Total CSA in Regression: The results state that Total CSA (the sum of all three muscles) showed the strongest single correlation with IKFS at 50% (r = 0.911). However, the regression model uses the individual CSAs. A brief sentence explaining why the individual measurements were favoured over the highly correlated Total CSA metric would provide helpful clarity.

Response:

Thank you for your contribution. In line with your comment, we have added a brief clarification in the Discussion to explain the rationale for including individual muscle CSAs rather than total CSA in the regression model.

[Line 266-268]

“*…Although total CSA showed the strongest correlation with IKFS, individual muscle CSAs were used in the regression model to identify muscle-specific contributions and to avoid overlap between the combined and individual measures…”*

**Comment 2.**

* Correlation Tests: In the "Statistical Analyses" section, the authors state that relationships were determined using "Pearson’s correlation coefficient (r)". However, the legend in Table III indicates that both Pearson's (α) and Spearman's (β) tests were used. The methodology should be updated to reflect the use of non-parametric tests.

Response:

We acknowledge that this was not clearly specified in the original manuscript and we thank the reviewer for highlighting it. In line with your suggestion, we have revised the Statistical Analyses section to clarify that both Pearson’s and Spearman’s correlation coefficients were used, depending on data distribution.

**Comment 3.**

Language and Typographical Corrections

Response:

We thank the reviewer for identifying these corrections. All grammatical and typographical errors highlighted by the reviewer have been corrected throughout the manuscript to improve clarity and readability.

**REVIEWER 2 SUGGESTIONS:**

**Comment 1.**

- It appears in the methods that US data (despite not for all parameters) was collected from three different points (25%, 50% and 75%). However, the study title only contains two values (25% and 50%). It can be assumed that the name was formed based on the results obtained, but more suitable alternative research titles are still suggested.

Response:

We appreciate your thoughtful suggestion. We have revised the title to better reflect the full scope of the study. The updated title is: “*Beyond Mid-Thigh: Ultrasound Assessment of Hamstring Muscle Architecture at Multiple Muscle Lengths in Relation to Strength.”*

This revised title reflects the inclusion of multiple measurement sites along the muscle length, rather than highlighting specific percentage locations. We believe this revised title more accurately represents the study design while maintaining clarity and readability.

We have also edited the abstract and introduction section to clarify the measurement sites.

[Line 25-27]

*“…to evaluate whether distal (25%) and proximal (75%) measurement sites differ in their ability to reflect muscle strength compared to the commonly used mid-thigh (50%) region.”*

[Line 39-41]

*“…At 75%, CSA showed a non-significant association with strength for the semimembranosus, while it could not be measured for the semitendinosus and biceps femoris long head due to their indistinguishable structure…”*

In addition, the Conclusions section has been modified to better reflect our findings:

[Line 48-49]

*“…In contrast, proximal measurements were limited by anatomical constraints and showed non-significant associations with strength. …”*

Similarly, the aim of the study in the Introduction has been revised to avoid presenting both regions as equivalent alternatives. These revisions ensure that the 75% site is now accurately framed as anatomically challenging and not suitable as a reliable alternative measurement site.

**Comment 2.**

- The abstract only includes CSA findings; there are no findings regarding other parameters (MT and PA). Therefore, the abstract could be reviewed.

Response: We thank your helpful observation. In the original version of the Abstract, we focused primarily on CSA findings, as these showed the strongest and most consistent associations with isometric strength. As a result, findings related to muscle thickness and pennation angle were not explicitly reported in order to maintain conciseness within the word limit. In line with the suggestion, we have revised the Abstract to also include these parameters. Specifically, we added the following sentence:

[Line 41-44]

*“…Muscle thickness showed significant correlations for the semitendinosus and semimembranosus (r = 0.702 and 0.698, respectively), whereas pennation angle demonstrated weaker and inconsistent relationships…”*

**Comment 3.**

- In the introduction section, have alternative points been investigated in other muscles? It's not mentioned at all. If so, such studies should be included.

Response: We are grateful for this suggestion. While the Introduction outlined the non-uniform nature of muscle architecture along the muscle length and emphasised the importance of considering alternative measurement sites, we acknowledge that evidence from other muscle groups was not explicitly mentioned to support this point. In response to your comment, we have added the following sentence to clarify this point:

[Line 87-89]

*“…in other muscle groups, such as the quadriceps and gastrocnemius, regional variations in muscle architecture along the muscle length have been previously investigated, highlighting the importance of site-specific assessments [17, 18]…”*

**Comment 4.**

- The reason why evaluation at different points in the hamstring muscle is needed is explained somewhat, but it should be explained in more detail clinically, and the gap in the literature that need clarification should be highlighted more clearly.

Response:

We thank you for raising this important point. In the Introduction, we had initially described the general importance of evaluating different measurement sites along the muscle length. However, we acknowledge that the clinical relevance and the specific gap in the literature were not sufficiently emphasised. In response to your comment, we have revised this section to clarify that the choice of measurement site may influence the clinical interpretation of muscle architecture and its association with strength. We have also explicitly highlighted the gap in the literature by stating:

[Line 89-95]

*“…From a clinical perspective, the choice of measurement site may influence how the relationship between ultrasound findings and muscle strength is interpreted. In this context, investigating alternative measurement sites is important, as distal regions are more prone to acute strain injuries, while proximal adaptations may reflect chronic or degenerative processes [19]. However, it remains unclear whether measurements taken from different regions of the hamstring muscles reflect muscle strength in the same way…”*

Furthermore, the Conclusion has been refined to reflect this consideration more clearly, as follows:

[Line 385-387]

*“…The findings also suggest that the relationship between muscle architecture and strength may vary depending on the measurement site....”*

**Comment 5.**

- The reason why the non-dominant side was preferred in the evaluation should be explained, and it is also recommended to include a reference.

Response:

We appreciate you bringing this point to our attention.

We have revised the Methods section to clarify the rationale for selecting the non-dominant extremity. A relevant reference has also been included to support this methodological choice.

Specifically, we added a statement indicating that

[Line 125-127]

*“ … on the non-dominant extremity to reduce the influence of limb dominance, given the well-documented strength asymmetries between limbs that may affect measurement consistency [21, 22]…”*

**Comment 6.**

- Although there is partial information about the individuals who performed the ultrasound measurements for inter-assessment, information should be provided regarding who performed the initial measurement at the beginning of the method, such as information on the training and experience in MSUS. It should also be specified whether the person(s) collecting the data were blinded to the research or not.

Response:

We appreciate you pointing out this aspect of the methodology. While information regarding the evaluators was provided in the context of the reliability analysis, we acknowledge that the description of the examiner responsible for the primary ultrasound measurements was not clearly stated at the beginning of the Methods section. In response to your comment, we have revised the Methods section to clearly specify the examiner responsible for the ultrasound measurements, including their training and experience, and to clarify that the examiners were not blinded to the study but were not involved in the strength assessment or statistical analysis.

[Line 127-130]

*“…All ultrasound measurements were performed by a sports physician with formal training in musculoskeletal ultrasound and over three years of experience in this field. A second experienced examiner independently performed repeated measurements for reliability analysis. The examiners were not blinded to the study but were not involved in the strength assessment or statistical analysis…”*

**Comment 7.**

-The description of the reliability analysis is unclear in the lines of 135 and 152. The statement refers to “inter-rater reliability,” whereas the use of repeated measurements appears to indicate intra-rater reliability. Please clarify this section of methods.

Response: We acknowledge that the description of the reliability analysis in the original version was not sufficiently precise and may have led to confusion.

In response to your comment, we have revised the Methods section to clearly distinguish between intra-rater reliability reported elsewhere for a single examiner and inter-rater reliability assessed in this study between two evaluators using the intraclass correlation coefficient (ICC).The revised statements now read as follows:

[Line 149-152]

*“ To assess inter-rater reliability, the intra-class correlation coefficient (ICC, two-way random effects model, absolute agreement) was calculated based on measurements obtained by two independent evaluators in the first 11 participants. The intra-rater reliability of the first physician is shown elsewhere [24].*

[Line 173-175]

*“… inter-rater reliability between the two evaluators was assessed using the intraclass correlation coefficient (ICC) in the first 11 participants [27].”*

**Comment 8.**

- There appears to be an inconsistency between the table title and the column heading. The table II title refers to “inter-rater” reliability, whereas the second column reports “intra-rater” coefficients. Please clarify whether the reported reliability analysis is inter-rater or intra-rater and revise the terminology accordingly.

Response:

We appreciate you pointing out this inconsistency. We acknowledge that the terminology used in this section was not sufficiently precise, which may have led to confusion between intra- and inter-rater reliability. We have revised the terminology to ensure consistency throughout the manuscript. Specifically, the reported reliability analysis corresponds to inter-rater reliability, and the column heading in Table II has been corrected accordingly.

**Comment 9.**

- Organizing the tables totally on each page would be more understandable for the reader.

Response:

We thank the reviewer for this helpful suggestion. We have revised the manuscript to improve the presentation of the tables by ensuring that each table appears in full on a single page wherever possible. This adjustment enhances readability and allows readers to more easily interpret the data without needing to navigate across pages.

We appreciate the reviewer’s input, which has helped improve the clarity of the manuscript.

**Comment 10.**

- Table 1 should use mm instead of cm and mm2 instead of cm2.

Response: We appreciate this helpful observation. Upon careful review, we identified that the units for muscle thickness (MT) were incorrectly reported as cm, whereas the values correspond to millimetres. This has now been corrected to mm in Table I. The units for cross-sectional area (CSA) have been retained as cm², as they accurately reflect the reported values and are consistent with commonly used conventions in the literature.

**Comment 11.**

- The results section titled "Correlations Between US Parameters and Strength" presents a rather complex flow for the reader. Instead of prioritizing high correlation levels, presenting results based on CSA, MT, and PA parameters would be more fluid and understandable.

Response:

We have revised the structure of the Results section to improve clarity and readability. Specifically, the findings are now presented according to ultrasound parameters (CSA, MT, and PA), rather than prioritising the strength of the correlations. This reorganisation allows for a more systematic and reader-friendly presentation of the results while maintaining the accuracy of the reported data.

**Comment 12.**

- Could SM at 50% on line 183 be ST at 50%? Because the previous sentence provides information about SM at 50%. It is recommended to check this sentence.

Response:

We appreciate this observation. We acknowledge that there was a wording issue in this sentence, which may have led to confusion. However, this was not a data error. In response to a previous comment, the Results section has already been revised and reorganised, and the wording has been clarified accordingly to ensure a clearer and more structured presentation of the findings.

[Line 208-209]

*“…The ST showed a significant association at 50% (P < 0.01; r = 0.802), while its correlation at 25% was not significant (P = 0.327; r = 0.231)…”*

**Comment 13.**

- Since the gender row in Table III does not contain correlation information, it is recommended that it not be included in this table.

Response:

We agree that the inclusion of the gender-related result in Table III was not appropriate, as this table is intended to present correlation analyses. The "Gender" row has been removed from Table III. The result has been retained in the main text of the Results section to clearly report the statistically significant difference in muscle strength between males and females.

**Comment 14.**

- The conclusion regarding the statement in the discussion, "These findings suggest that, contrary to the conventional focus on the mid-thigh (50%) region, distal measurement sites (25%) can also provide valuable insight into muscle strength," is thought-provoking; this conclusion can only be drawn for CSA at 25%, as data collected from the 50% region are more prominent in this study for other parameters.

Response:

We appreciate this insightful comment. We agree that the original statement may have been overly broad, as the findings supporting distal measurement sites were primarily related to CSA rather than all ultrasound parameters. We have revised this sentence to clarify that the observed relevance of distal measurements (25%) is particularly associated with CSA findings.

The revised sentence now reads as follows:

[Line 268-271]

*“…These findings indicate that the relationship between muscle architecture and strength varies along the muscle length. In addition to the focus on the mid-thigh (50%) region, distal measurement sites (25%), particularly for CSA of the semimembranosus and total CSA, may also provide valuable insight into muscle strength…”*

**Comment 15.**

- The paragraph starting at line 233 already includes information in the introduction; it's a paragraph that only presents the conclusion without containing sufficient discussion and should be reviewed.

Response:

Thanks for highlighting this point. We edited the related part as follows:

[Line 278-294]

*“…In the present study, isometric hamstring strength was positively correlated with the CSA and MT of the SM, ST, and LBF, consistent with the general principle that physiological CSA is related to a muscle's force-generating capacity [30, 31]. The physiological basis for this relationship is that a larger CSA reflects a greater number of sarcomeres, and force production scales with the amount of contractile tissue available to generate tension [30]. Although similar associations have been reported in older adults and clinical populations, our results extend this relationship to a younger, healthy cohort, indicating that muscle architectural characteristics play a consistent role in strength across populations [3, 4]. However, in clinical conditions such as hamstring strains, where pain and tenderness are common, muscle strength may not be objectively assessed using HHD or isokinetic testing due to painful contractions [32, 33]. Utilising US-based indirect strength measurement may provide some insight into muscle strength “without pain,” which is important when returning to sports following hamstring injury [10]. However, it should be noted that muscle strength is multifactorial, and neural activation, tendon properties, and muscle fibre composition may also contribute to the observed variability. Therefore, while CSA and MT appear to be important determinants, they should be considered alongside other neuromuscular factors when interpreting hamstring strength capacity.…”*

**Comment 16.**

- The paragraph starting on line 247 presents good information, but it lacks sufficient discussion of the research findings. It could be revised by reviewing the results of similar studies (especially those involving other muscles) and the discussion sections in general.

Response:

Thanks for underpinning the necessary editing in this paragraph. Based on your suggestions, we edited the text as follows:

[Line 295-314]

*“…The balance of muscle strength between the anterior and posterior thigh muscles is a fundamental for maintaining motor control and functional performance. The observed relationship between hamstring strength and US-derived architectural parameters (CSA and MT) in this study suggests that morphological characteristics provides valuable insight into functional muscle capacity. Similar associations have been also reported in other muscle groups, such as the quadriceps, where greater muscle thickness and CSA are linked to higher force production [12, 13, 33]. This supports the notion that muscle architecture plays a central role in determining strength across different anatomical regions. In neurological conditions, where muscle weakness, spasticity, and impaired motor control coexist, these relationships may become more complex [34]. Interventions used to restore hamstring–quadriceps balance, including stretching exercises, surgical lengthening, or tone-reducing interventions (e.g. selective dorsal rhizotomy or botulinum toxin injection), may further decrease muscle strength [30, 35]. Therefore, accurate assessment of hamstring strength is essential but challenging in these populations. In this context, US muscle architecture parameters may offer a non-invasive approach to estimate isolated muscle in neurologically affected patients. Although the current study evaluated US parameters in healthy patients, the findings provide a necessary baseline for understanding how architectural parameters relate to isolated muscle force. Therefore, prior to investigating the relationship between isometric strength and US-based parameters of an isolated muscle group in a complex disease, it is imperative to understand these relationships in able-bodied individuals.…”*

**Comment 17.**

- The discussion is generally quite long, and the research findings are not discussed sufficiently; there is a loss of focus due to the inclusion of various topics. It is recommended that the discussion section be reviewed and revised.

Response:

We thank the reviewer for this valuable feedback. In response, we have carefully revised the Discussion section to improve its clarity, focus, and balance. Specifically, we have reduced redundancy and removed less relevant or tangential content to enhance conciseness. Greater emphasis has been placed on the direct interpretation of the study’s key findings, particularly the relationships between ultrasound-derived parameters and muscle strength, as well as the implications of measurement site differences. Additionally, we have streamlined the structure to ensure a clearer and more logical flow between results, interpretation, and clinical relevance.

These revisions have improved the overall readability and strengthened the focus of the Discussion.

**Comment 18.**

-Limitations may include the lack of examination of muscle strength values related to isokinetic muscle assessment, and it is recommended that further studies be conducted in this research field.

Response:

Thanks for the comment. We added a paragraph in the limitation section to address this point.

[Line 363-368]

*“…Another limitation is that muscle strength was assessed only under isometric conditions, without the inclusion of isokinetic measurements. Isokinetic assessment could provide additional insight into muscle performance across different angular velocities and may better reflect dynamic functional capacity. Age‑related changes in muscle architecture and neuro-muscular function, differences in training status, and variations in body composition may substantially alter the relationships observed here. …”*
