# Supplementary material for: Beyond mid-thigh: Ultrasound assessment of hamstring muscle architecture at multiple muscle lengths in relation to strength
Source: PLoS One. 2026 Oct 27;21(10):e0361496. doi: 10.1371/journal.pone.0361496 (PMC13712582; doi:10.1371/journal.pone.0361496)
Supplement: Supplementary file 3 [file pone.0361496.s004.docx]

We would like to thank the Editor and Reviewers for their careful evaluation of our manuscript and for the constructive comments that helped improve its quality and clarity. We are pleased that the revisions have addressed the major concerns raised during the previous review round.

In this revision, we have addressed all remaining comments from Reviewer #2. Specifically, we have: (1) removed the unintended “Gender” row from Table III and revised the table title accordingly, (2) shortened and refocused the Discussion by substantially reducing the section concerning neurological conditions and related interventions to better align with the primary findings of the study, and (3) replaced the term “healthy patients” with “healthy participants” to accurately describe the study population. In addition, we carefully reviewed the revised manuscript and corrected a couple of wording, grammar, and language issues to improve overall clarity, consistency, and readability.

We are grateful for the reviewers’ positive assessment of the revised manuscript and their valuable suggestions, which have helped improve its clarity and scientific focus. We believe that the manuscript has been strengthened substantially and hope it is now suitable for publication.

Thank you for your consideration.

Sincerely,

**Ali Cihan Dagli**

PhD Physiotherapy

School of Health Sciences

The University of Nottingham, UK

alyad2@nottingham.ac.uk

**REVIEWER 1**

We sincerely thank the reviewer for the positive assessment of our revised manuscript and for recognising the efforts made to address the previous comments. We appreciate the reviewer’s acknowledgement of the clarification regarding the anatomical limitations of the 75% measurement site, the inclusion of multicollinearity diagnostics, the expanded discussion concerning isometric versus eccentric muscle function, and the provision of the raw dataset. We are grateful for the recommendation for publication.

**REVIEWER 2 SUGGESTIONS**

We thank the reviewer for the careful re-evaluation of the manuscript and for the helpful suggestions. The manuscript has been revised accordingly.

**Comment 1.**

First, although the authors stated that the “Gender” row has been removed from Table III, this row is still present in the revised manuscript. Please remove the “Gender” row and revise the table title accordingly.

Response:

Thank you for identifying this oversight. The "Gender" row has now been removed from Table III, and the table title has been revised accordingly to accurately reflect the remaining contents of the table.

**Comment 2.**

Second, the paragraph discussing neurological conditions, muscle weakness, spasticity, and interventions for restoring hamstring–quadriceps balance appears overly lengthy and somewhat tangential to the main findings of the present study. The Discussion should be reviewed again and this section shortened to maintain focus on the study findings and avoid unnecessary digression.

Response:

We appreciate the reviewer’s observation and agree that this section was overly detailed relative to the primary objectives of the study. The paragraph has been substantially shortened and refocused on the main findings of the present study. Extensive discussion regarding neurological conditions, muscle weakness, spasticity, and interventions aimed at restoring hamstring-quadriceps balance has been removed, while retaining only a brief statement highlighting the potential relevance of ultrasound-derived architectural measures for future clinical research. This revision improves the focus and overall readability of the Discussion.

[Line 293-306]

*“…The balance of muscle strength between the anterior and posterior thigh muscles is a fun-damental factor for maintaining motor control and functional performance. The observed relationship between hamstring strength and US-derived architectural parameters (CSA and MT) in this study suggests that morphological characteristics provides valuable insight into functional muscle capacity. Similar associations have been also reported in other muscle groups, such as the quadriceps, where greater muscle thickness and CSA are linked to higher force production [12, 13, 34]. This supports the notion that muscle architecture plays a cen-tral role in determining strength across different anatomical regions. In this context, US muscle architecture parameters may offer a non-invasive approach to estimate isolated muscle in neurologically affected patients. Although the current study evaluated US parame-ters in healthy participants, the findings provide a necessary baseline for understanding how architectural parameters relate to isolated muscle force. Therefore, establishing the rela-tionship between isometric strength and US-derived muscle architecture in healthy individu-als is an essential before examining these associations in clinical populations.…”*

**Comment 3.**

Finally, the statement “Although the current study evaluated US parameters in healthy patients” should be revised, as the participants in the present study were healthy young adults rather than patients. “Healthy participants” or “healthy young adults” would be more appropriate.

Response:

Thank you for this correction. The term “healthy patients” has been replaced with “healthy participants” throughout the manuscript to accurately reflect the study population. In addition, we carefully reviewed the revised manuscript and corrected a couple of other wording, grammar, and language issues to improve overall clarity, consistency, and readability.
